# Spontaneous alloparental care of unrelated offspring by non-breeding *Amphiprion ocellaris* in absence of the biological parents

**DOI:** 10.1038/s41598-020-61537-7

**Published:** 2020-03-12

**Authors:** Elizabeth Phillips, Ross DeAngelis, Joseph V. Gogola, Justin S. Rhodes

**Affiliations:** Department of Psychology, Beckman Institute for Advanced Science and Technology University of Illinois at Urbana, Champaign 405 N. Mathews Ave Urbana, IL, 61801 USA

**Keywords:** Neuroscience, Social behaviour

## Abstract

Many species display alloparental care, where individuals care for offspring that are not their own, but usually the behavior is contingent on the individual receiving some direct or indirect benefit. In anemonefish, after removing the breeding male, non-breeders have been observed providing care for eggs they did not sire and which are not kin. Previously this behavior was interpreted as coerced by the female. The purpose of this study was to test the alternative hypothesis that the alloparental care occurs spontaneously without prodding by the female. Groups of *Amphiprion ocellaris* (male, female and non-breeder) were maintained in the laboratory and behavior monitored after removing the male and both the male and female. Non-breeders began to care for eggs after male removal and further increased parental care after male and female removal. Level of care was not as high as experienced males, but additional experiments showed performance increases with experience. In a separate experiment, non-breeders were placed alone in a novel aquarium and eggs from an established spawning pair were introduced. Approximately 30% of the fish displayed extensive fathering behavior within 90 min. Taken together, our results demonstrate that fathering behavior in *A. ocellaris* occurs spontaneously, independent of paternity or kinship.

## Introduction

Alloparental care, where individuals care for offspring that are not their immediate descendants occurs with reasonable frequency in nature^1–6^. However, usually the behavior is actively reinforced by the breeding individuals or contingent on their presence. For instance, in the cooperative breeding cichlid, *Neolamprologus pulcher*, unrelated subordinates assist the dominant pair in reproduction, otherwise they would be chased out of the territory by the larger breeding pair^5,7^. Similarly, in anemonefish *Amphiprion clarkii*, subordinate, non-breeders care for eggs they did not sire and which are not kin, and the behavior is explained as being coerced by the presence of the dominant female^8^. However, anemonefish have a peculiar life history which may have provided the right context for the appearance of unconditional, spontaneous fathering behavior independent of paternity or experience.

Anemonefish, or clownfish, are an iconic group of coral reef fish, beloved by the aquarium hobby, and as such their life history has been studied for decades and extensively reviewed elsewhere^9,10^. Anemonefish, such as *A. clarkii*, and especially *A. ocellaris*, typically live in small groups consisting of one alpha female and one beta male which reproduce together exclusively (i.e., genetic monogamy) plus zero to several gamma non-reproductive subordinates^11,12^. The reproductively suppressed gamma individuals are sexually immature^13,14^. However, they can rapidly acquire male reproductive potential if the male is removed^15^. These gamma individuals are tolerated by the breeding pair and maintain their own dominance hierarchy below the breeders^16^. They do not normally provide care towards the eggs and are usually chased away from the nest especially by the male. It was estimated that the non-breeders have little impact on the breeder’s fitness, even when one of the breeders is displaced because potential mates from neighboring territories are easily recruited^17^. Although the non-breeders have zero share in the current reproductive effort, it has been hypothesized that they stay because they need the protection of the anemone and have the potential to reproduce in the future depending on the fate of the current reproductive pair and their position in the dominance hierarchy^18^. If the beta male is displaced, the highest-ranking non-breeder takes his place and becomes the reproductive male. If the female is displaced, the beta male changes sex and becomes the alpha female, and gamma becomes beta^14,15,19–24^. All the individuals are dependent on the host anemone for protection from predators and because the anemone hosts are typically spatially separated, groups are reproductively isolated from one another^25,26^. Moreover, anemonefish have a planktonic larval phase where recruitment to a specific anemone is a rare and highly stochastic event^27^ making it exceedingly unlikely that any two members of the same group are kin, as shown by fingerprinting studies^28,29^.

Most fish will eat eggs that are not their own if given the chance because eggs are a rich source of nutrition^30–32^. Therefore, the observation that *A. clarkii* non-breeders care for eggs that they did not sire after the male is experimentally removed from the territory is surprising and intriguing^8^. The male removal study by Yanagisawa and Ochi^8^ was conducted in the field, specifically in the temperate waters off the coast of Japan. They removed males from 33 groups, and in 17 of the 33 observed a non-breeder caring for eggs that were not their own and not kin. They further reported observing the female “butting” or “nudging” the non-breeder, which they interpreted as coercing the non-breeder to care for her eggs, rather than the non-breeder voluntarily choosing to care for them on his own. Individuals that threaten or challenge the dominant female or male’s status typically elicit aggression and are evicted from the anemone^33^. In this way, the non-breeder would benefit by caring for the unrelated eggs by avoiding injury or displacement. To date, no studies have yet examined if anemonefish continue to provide parental care towards eggs they did not sire without the presence of the female to coerce them into performing the behavior.

The goal of this study was first to determine the extent to which alloparental care by non-breeders occurs in the iconic species of anemonefish, *A. ocellaris* (Experiment 1). We hypothesized non-breeders would care for eggs they did not sire and which are not kin when the male is removed, similar to *A. clarkii*^8^ since they are closely related species and share a similar life history^34^. Second, we wanted to determine whether the alloparental care persists if the non-breeder is left alone with the eggs, without the possibility of the female’s coercion (Experiment 1). We hypothesized that it would persist, even without the female, given that in nature elimination of the male while the brood is present would be a relatively rare event, and hence not cost that much in terms of overall fitness. Third, we wanted to determine whether the stepfather would devote similar attention to his surrogate brood as his own brood (Experiment 2). We hypothesized they would, consistent with the idea fathering occurs unconditionally and independent of paternity. Fourth, we wanted to assess whether fathering performance improves upon successive broods since in Experiment 2, by the time the stepfathers cared for their own brood, they had 2 previous experiences caring for a surrogate brood. We hypothesized fish would display improved performance because of experience rather than because of favoritism toward their genetic offspring (Experiment 3). Fifth, we wanted to determine whether non-breeders, taken from a group-housing condition that had never witnessed nor ever taken part in a spawning event, would care for eggs if they were placed into their territory. Again, we expected they would care for offspring that are not their own based on the hypothesis that fathering is a hard-wired behavior in the species and occurs independent of paternity or kin, needing only the egg stimulus and the opportunity.

## Methods

All experimental procedures involving animals were conducted in accordance with the regulations and guidelines set forth by the United States Department of Agriculture (USDA) and the Association for Assessment and Accreditation of Laboratory Animal Care (AAALAC). All the experimental procedures were approved by the Institutional Animal Care and Use Committee (IACUC) at the University of Illinois.

### Animals and husbandry

A total of 55 *A. ocellaris* were used in this study. The fish were either directly obtained from Oceans Reefs and Aquariums (ORA; Fort Pierce, Florida) or bred in-house from a cross between an ORA fish and wild caught fish obtained through the pet trade (F1). Twenty-gallon tall (24” × 12” × 16”) and 25-gallon cube (18” × 18” × 18”) aquaria were used to house fish. Aquaria were integrated via plumbing to a large circulating filtration system. All tanks contained a terra-cotta pot (6” or 8” diameter) for the nest site where eggs are deposited. Some tanks also contained sand and live rock. In experiment 4, standard 10 gallon tanks were used to isolate non-breeders for 2 weeks prior to egg introductions. Water from the main systems were used to fill these 10 gallon tanks and sponge filters and heaters were used to maintain water quality throughout the experiment. Conditions were set to mimic the natural environment with a pH between 8.0–8.4, temperature range of 79–82 °F, photoperiod of 12:12 (lights on at 0700 hrs and off at 1900), and specific gravity of 1.026. Fish were fed twice daily with a mix of New Life Spectrum Marine Formula (Homestead, Florida) and Golden Pearls (from Brine Shrimp Direct, Ogden, Utah).

### Experimental design

#### Experiment 1- Male and female removal

**Figure 1:**
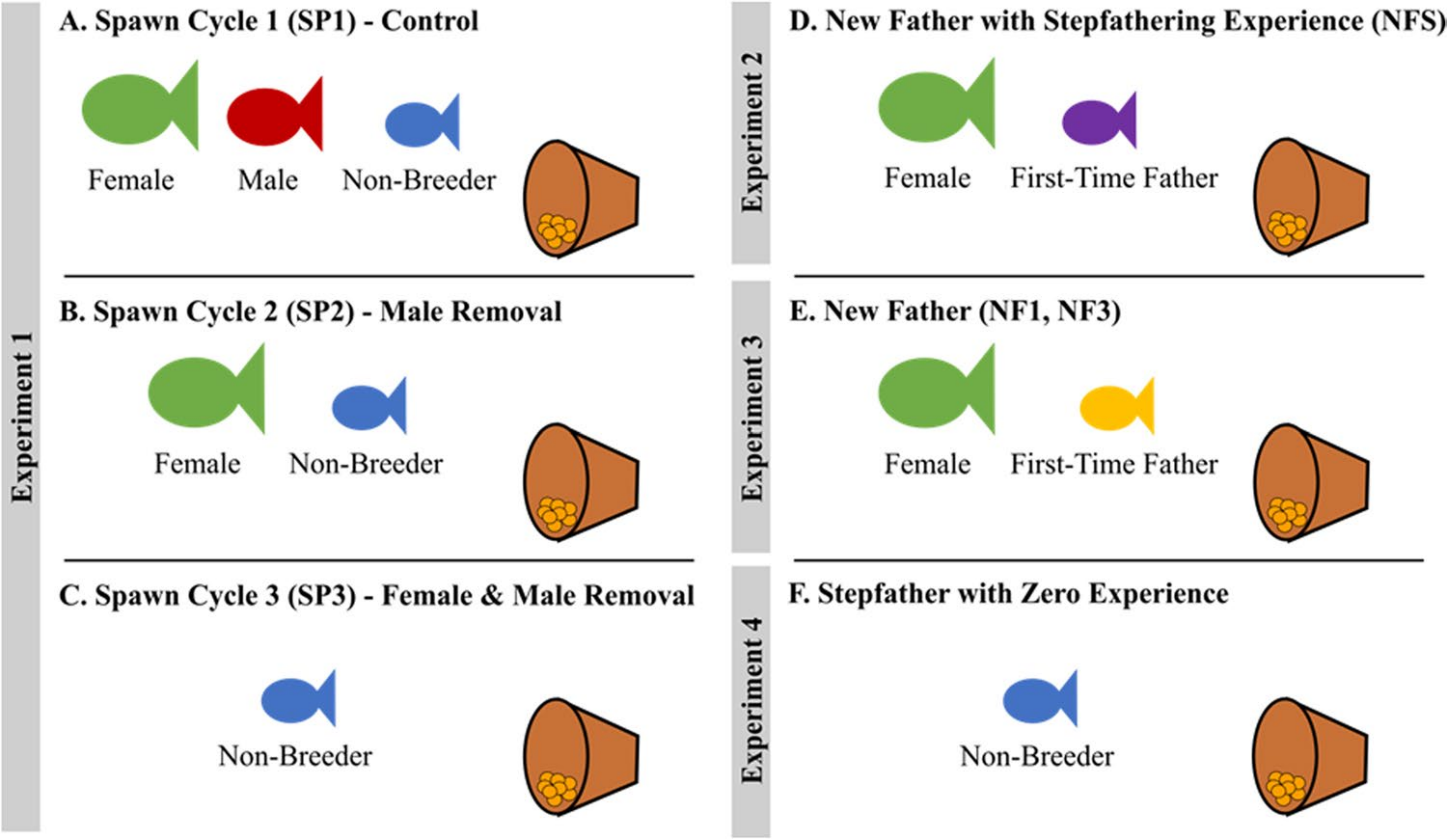
Experimental design. (**A**) Experiment 1 consisted of 3 spawn cycles (9 groups of 3 fish each). Spawn cycle 1 (SP1) was the Control condition where the male, female and non-breeder were present. (**B**) In spawn cycle 2 (SP2), the male was removed. (**C**) In spawn cycle 3 (SP3) both the male and female were removed, leaving only the non-breeder to care for the eggs. (**D**) Experiment 2 consisted of subsample of groups from Experiment 1 (5 groups of 2) which experienced an additional manipulation after the experiment was completed. The male was permanently removed and the non-breeder allowed to fertilize his own first brood of eggs with the female. This group is referred to as new father with step-fathering experience (NFS). (**E**) Experiment 3 included a new group of 6 pairs of fish. Each pair consisted of an experienced female, and a new male that had never reproduced before, taken from a stock tank containing a large number of non-breeding fish. These individuals were allowed to spawn 3 times. Behavioral data were analyzed for the very first (NF1) and third time (NF3) the fish spawned. (**F**) Experiment 4 included a new group of 16 non-breeders, approximately 1-year old, taken from the stock tank, which had never witnessed a spawning event. They were singly housed for 2 weeks before their terra cotta pot was replaced with one containing a batch of eggs taken from a breeding pair in the colony.

The purpose of this experiment was to determine the extent to which the non-breeder would display alloparental care if the male and female were removed from the territory. The experiment included 9 aquaria with 3 fish in each (27 fish total), that had been established for at least one year. The 3 fish consisted of a reproductive male, a reproductive female, and a subordinate non-breeder with the breeding pair spawning regularly (at least once per month for at least three months). Fish were monitored daily between the hours of 11:00 AM and 2:30 PM to check for eggs. An entire spawn cycle from the time the eggs are laid to the time they hatch takes between 7 to 9 days. The day when newly laid eggs were found was denoted as Day 1 of the spawn cycle. On Day 2, the terra cotta pot was removed from the tank for a brief period (less than 2 minutes) so the eggs could be photographed. On days 3–6, a 15-min video was taken to record behavior of each individual in the triad (see Behavioral Analysis below). Of this 15 min video, only the last 10 min were analyzed to allow fish time to acclimate after setting up the video camera in front of their aquarium. During behavioral recording, the room was cleared of people to ensure movement outside of the tanks did not affect fish behavior. As shown previously, parental care behavior is consistent across days and across spawn cycles, and the 10 min window is sufficient to capture reliable individual variation^35^. After video recording on Day 6, eggs were photographed again by briefly removing the terra cotta pot (see Egg Loss Analysis below). This entire process was repeated for 3 spawn cycles. Control (spawn cycle 1): Fish remained undisturbed to record baseline behavior. Male Removal (spawn cycle 2): The male was removed on Day 3 immediately before the video session, and was returned on Day 6 after photographing eggs. We expected the behavior of the non-breeder and the female to change immediately in response to removing the male and persist for the subsequent testing days. Male and Female Removal (spawn cycle 3): Both the male and female were removed, following the same time-line as spawn cycle 2 (Fig. 1A–C).

#### Experiment 2- First-time fathers with prior step-fathering experience

The purpose of this experiment was to determine whether the stepfather would devote similar attention to his surrogate brood as his own brood. A subsample of groups from Experiment 1 (5 groups) experienced an additional manipulation after the experiment was completed. First-time fathers after prior step-fathering experience: The male was permanently removed, and the previous non-breeder was allowed to fertilize his own first brood of eggs (Fig. 1D). The previous non-breeder took between a 7 days and 2 months to spawn (mean was 32.6 days ± 22 SD). Data collection proceeded as described for Experiment 1, spawn cycle 1.

#### Experiment 3- First-time and third-time fathers

The purpose of this experiment was to determine whether fathering performance (i.e., number of parental care acts and percent egg survival) improves upon successive broods since in Experiment 2, by the time the stepfathers cared for their own brood, they had 2 previous experiences caring for a surrogate brood. Experiment 3 included 6 new pairs (12 fish total). The pairs consisted of an experienced female, and a new male that had never reproduced before, taken from a stock tank containing a large number of non-breeding fish approximately 1-year old. Newly-formed pairs spawned between one week and two months after pairs were formed (mean was 22.0 days ± 9.0 SD). First-Time Father: The very first time the male fertilized and cared for eggs was recorded. Data collection proceeded as described for Experiment 1. Third-Time Father: Exactly the third-time the male fertilized and cared for eggs was recorded using the same data collection procedure (Fig. 1E).

#### Experiment 4- First-time stepfathers

Non-breeders taken from a stock tank containing a large number of fish that were approximately 1-year old were placed individually in a 10 –gallon aquarium with a terra cotta pot and left alone for 2 weeks. Following the acclimation period, the terra cotta pot was replaced with a pot containing eggs on day 2 of development taken from the colony. A video camera was placed in front of the tank and the fish was left alone for 90 min. The last 20 min of video feed were analyzed for percent time in nest and number of nips and fans (Fig. 1F).

### Behavioral analysis

The purpose was to quantify levels of parental care and aggression in response to the social manipulations. Videos were scored for parental care and other social behaviors using JWatcher behavioral event recording software following previous reports^35–38^. In experiments 1–3, a total of 40 min of video was scored (10 min x 4 days) for each individual per spawn period (treatment). Data are shown as mean number of behaviors per 10 min. In experiment 4, the last 20 minutes of a 90 min trial was scored. Behaviors scored included number of parental acts (sum of nips and fans on the eggs), number of aggressive acts (charging, biting or dominance displays), duration in the nest, and number of approaches towards the nest (see descriptions in Table 1). Approaches to the nest were measured to evaluate the motivation of the non-breeder towards entering the nest in the presence and absence of the female. Each behavior was scored by the same investigator who was blind to the experimental conditions except where unavoidable such as when fish were removed from aquaria.

**Table 1:** Description of scored behaviors.

Behavior	Description
Parental care	Nipping, which refers to mouthing near the eggs, and fanning, which involves a full body shake, or using pectoral fins to fan over or around eggs.
Time in the nest	Percentage of time when whole body is in the terra cotta pot.
Aggression	Darting or intentional quick motion towards another individual, or dominance displays. Can sometimes result in biting.
Approach	Intentionally swimming close to the pot (near the entrance), but not entering the nesting area

### Egg loss analysis

The total number of eggs present on Days 2 and 6 of each spawn cycle was counted using the Cell Counter plugin in ImageJ software. Individual eggs were counted by hand by a trained observer, blind to experimental treatments. Initial pictures were taken on Day 2 instead of Day 1 so that eggs could be identified as either fertilized (brown) or unfertilized (orange). Only fertilized eggs were counted, since unfertilized eggs are usually eaten by the father and are not indicative of offspring survival due to parenting. To determine baseline egg survival in absence of any parenting, 3 batches of eggs were analyzed from an additional 3 established spawning pairs. After these pairs were observed with a fresh batch of fertilized eggs on Day 2, all individuals in the tank were removed on Day 3 and returned on Day 6 to mimic the timeline used to test step-fathering of the non-breeders. Videos were not recorded during this condition, but pictures were taken of the eggs on Day 2 and Day 6. Egg loss was quantified as a percentage: 100×(number of eggs on Day 2 minus number on Day 6)/number on Day 2.

### Statistical methods

Data were analyzed using SAS (version 9.3) Proc Mixed and R (version 3.5.1) statistical software. Data were considered normally distributed if the residual distribution showed skewness between −1 and 1 and kurtosis between −2 and 2. Homogeneity of variance between groups was evaluated using Levene’s test. Experiment 1: To compare levels of parental care between males, females and non-breeders under baseline conditions when all three fish were present, total number of parental behaviors and duration in the nest were analyzed separately by mixed-effects 1-way ANOVA (with three levels; male, female, non-breeder). Tank was entered as a random effect to account for dependency between individuals in a tank. Significance of the random effect was established by performing a chi-square test on the difference in the log likelihood between models with and without the random effect. A similar mixed-effects 1-way ANOVA was performed to compare levels of parental care, duration in the nest and number of approaches toward the nest displayed by non-breeders during the three conditions: both male and female present, male absent, both male and female absent. To determine whether females changed their levels of parental care behaviors after males were removed, total parental behaviors and duration in the nest were analyzed a similar way except with only 2 levels (males present, males absent), equivalent to a paired t-test. Aggression was compared between males, non-breeders and females across the first 2 spawn cycles a similar way. This analysis had 5 levels: male spawn cycle 1, female spawn cycle 1, female spawn cycle 2, non-breeder spawn cycle 1, non-breeder spawn cycle 2. To determine how well the non-breeder’s parental care effort compared to the established males, total parental behaviors, and duration in the nest were compared using a mixed-effects 1-way ANOVA with three levels: males spawn cycle 1, non-breeders spawn cycle 2, non-breeders spawn cycle 3. Experiment 2 and 3: The same fathering performance measures used in Experiment 1 were re-analyzed with additional data. A similar mixed-effects 1-way ANOVA was used except with an additional 3 levels for a total of 6 levels: males, non-breeders spawn cycle 2, non-breeders spawn cycle 3, first-time fathers after step-fathering twice (Experiment 2), first-time and third-time fathers with no prior experience (Experiment 3). To compare quality of parental care between males with different levels of experience and females, total number of parental behaviors was compared between groups by mixed-effects analysis of covariance with duration in the nest entered as the covariate and 5 groups representing different amounts of experience caring for broods: males (spawn cycle 1), females (all spawn cycles collapsed), first-time fathers, stepfathers (spawn cycles 2 and 3 collapsed), and third-time fathers (collapsed across first-time fathers with twice step-fathering experience and 3rd time fathers, since for both groups it was their 3rd experience caring for eggs).

To determine whether changes in female parental care during step-fathering (in Experiment 1) is due to the disruption in the dominance hierarchy or inexperienced fathering, total number of parental care behaviors and duration in the nest were compared across groups using mixed-effects 1-way ANOVA in females only. A similar analysis was conducted in Experiment 1 comparing females with and without the male present. This analysis added three more groups: females with first-time fathers, third-time fathers, and first-time fathers after two batches of step-fathering. Finally, to determine whether egg survival during step-fathering is any different from first-time fathers, percent of eggs lost was analyzed using mixed-effects 1-way ANOVA with all 6 conditions: baseline (all three fish present), male removal, male and female removal, first-time fathers after step-fathering twice, first-time fathers, third-time fathers.

Fisher’s Least-Significant Difference (LSD) tests were conducted following a significant ANOVA result to identify which pair-wise differences between means were significant. Total number of parental care behaviors (sum of nips and fans) was log transformed (after adding 1 to the values to avoid undefined values) to equalize variance across treatment groups. Similarly, non-breeder approaches to nest were square-root transformed for the same reason. In experiment 4, Pearson’s correlation was used to estimate the correlation between percent time in the nest and total number of parental care behaviors, log transformed as described above. A p-value less than 0.05 was considered statistically significant. Measures are reported as mean ± standard error of the mean.

## Results

### Experiment 1- male and female removal

#### Non-breeders perform zero parental care when the breeding pair is present

**Figure 2:**
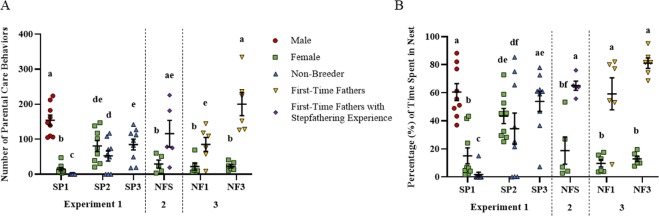
Non-breeders care for non-kin after male and female removal. (**A**) Total number of parental care acts (sum of nips and fans) shown by treatment group. (**B**) Percent time spent in the nest. Individual data points are shown, along with means as horizontal lines bounded by standard error bars. Different letters denote significant post-hoc differences between means. For the x-axis legend see Fig. 1.

During the Control condition, when all 3 fish were present, the male performed over 90% of the total parental acts directed toward the eggs, and females less than 10% (Fig. 2A, spawn cycle 1, first set of 3 bars). The non-breeders performed zero parental care behaviors. This was indicated by significant mixed-effects 1-way ANOVA comparing levels of care in males, females and non-breeders at spawn cycle 1 (F_2,16_ = 245.5, p < 0.0001). Post hoc tests indicated all pairwise differences were significant (all p < 0.0001). Time in nest reflected the same difference (F_2,16_ = 40.0, p < 0.0001) with all post hoc pair-wise comparisons significant (all p < 0.05; Fig. 2B). The random effect of tank was not significant in either analysis, nor was it significant for any of the analyses below.

#### Non-breeders provide care for eggs they did not sire when the breeding pair is absent

In most cases (6 out of 9 groups), after the male was removed, the non-breeder began to care for the eggs. In all cases, (9 out of 9 groups), after the male and female were removed, the non-breeder cared for the eggs. Levels of parental care provided by the non-breeder significantly increased from spawn cycle 1 to 2, from zero to a mean of 52 (± 5.0 SE) parental care acts when the male was removed. Number of parental acts increased by an additional 60% from spawn cycle 2 to 3, when both the female and male were removed (Fig. 2A; comparing blue bars between spawn cycles 1,2, and 3). This was indicated by a significant mixed-effects 1-way ANOVA for parental care among non-breeders in spawn cycles 1–3 (F_2,16_ = 26.3, p < 0.0001). Post hoc tests indicated all pair-wise differences were significant (all p < 0.03). Results for duration in the nest were similar (Fig. 2B). The ANOVA was significant (F_2,16_ = 13.1, p = 0.0004), and all pairwise tests were significant (both p < 0.005) except between spawn periods 2 and 3 (p = 0.09).

#### Females elevate care when male is removed, and increase aggression directed toward the non-breeder

**Figure 3:**
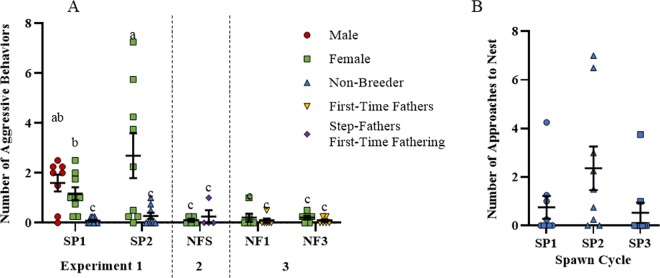
Female aggression toward non-breeders occurs in parallel with non-breeder approaches to the nest. (**A**) Total number of aggressive acts (sum of charges, bites and displays) shown separately for each treatment. (**B**) Total number of approaches by the non-breeder to the nest during spawn periods 1–3. Individual data points are shown, along with means as horizontal lines bounded by standard error bars. Different letters denote significant post-hoc differences between means. For x-axis legend see Fig. 1.

Number of parental acts displayed by the female towards the eggs (nips and fans) increased 5.7-fold in spawn cycle 2 when the male was removed, as compared to spawn cycle 1 when all 3 fish were present (F_1,8_ = 39.6, p = 0.0002, Fig. 2A, compare green symbols between spawn cycles 1 and 2). Similarly, female duration in the nest increased from 15% to 43% in spawn cycle 1 to 2, respectively (F_1,8_ = 25.3, p = 0.001, Fig. 2B). In addition to providing greater care and spending more time in the nest, females displayed approximately 2 more aggressive acts toward the non-breeder in spawn cycle 2 than 1 as indicated by a significant mixed-effects 1-way ANOVA on total aggressive acts across all individuals in spawn cycles 1 and 2 (F_4,31_ = 6.7, p = 0.0005; Fig. 3A, compare green bars between spawn cycles 1 and 2). Post hoc tests indicated elevated aggression from spawn cycle 1 to 2 for females (p = 0.01), elevated aggression in males and females relative to non-breeders in spawn cycles 1 and 2 (both p < 0.03), and no differences in aggression between males and females. The heightened aggression of females in spawn cycle 2 occurred in parallel with an increased number of non-breeder approaches to the nest (Fig. 3B). The mixed-effects 1-way ANOVA for number of approaches by the non-breeder was not significant (F_2,16_ = 2.8, p = 0.09; Fig. 3B). However, post hoc pair-wise tests indicated significantly more approaches in spawn cycle 2 as compared to 3 (p = 0.04). The difference between spawn cycle 1 and 2 did not reach statistical significance (p = 0.09). No difference was detected between spawn cycles 1 and 3.

#### Initial step-fathering behavior of non-breeders is inferior to experienced males

Non-breeders displayed 35% as many parental acts as males in spawn cycle 2 when the male was removed, and 56% as many in spawn cycle 3 when both the male and female were removed. This was indicated by a significant mixed-effects ANOVA considering only males (spawn cycle 1) and non-breeders in spawn cycles 2 and 3 (F_2,16_ = 12.6, p = 0.0005; Fig. 2A, comparing the red symbols in spawn cycle 1 to the blue symbols in spawn cycles 2 and 3). All post hoc pairwise differences were significant (p < 0.004) except between non-breeders in spawn cycles 2 and 3 (p = 0.14).

### Experiment 2- First-time fathers with prior step-fathering experience and Experiment 3-First and third-time fathers

#### Fathering behavior improves with experience and explains the inferior performance of stepfathers relative to experienced males in Experiment 1

 To evaluate whether the inferior fathering behavior by the non-breeders in Experiment 1 was a result of lack of experience by the non-breeders or decreased effort because the eggs were not their own, we let a subset of the non-breeders (5 of the 9) have their own brood for the first-time (by removing the male permanently) and evaluated their behavior (Experiment 2). Note that their first brood is actually their third experience caring for eggs, just the first-time the eggs are their own. Therefore, for comparison, we made 6 new groups by pairing non-breeders from a group housed tank with experienced females and evaluated their behavior the first and third time they had their own brood (Experiment 3).

Experienced males from the first experiment displayed 1.8-fold greater number of parental acts than first-time fathers. Levels of care by first-time fathers and stepfathers were similar (Fig. 2A, compare the blue symbols in spawn cycles 2 and 3 to the first set of yellow symbols above NF1). Moreover, fathering behavior improved from the first to the third time, such that levels of parental care by third-time fathers was similar to experienced males (Fig. 2A, compare last set of yellow symbols above NF3 to red symbols above SP1). This was indicated by a significant ANOVA comparing fathering among males, stepfathers during spawn cycles 2 and 3, first-time and third-time fathers, and first-time fathers with step-fathering experience (F_5,25_ = 6.7, p = 0.0005). Post hoc tests indicated that all pairwise differences between means were significant (all p < 0.05) except for the following: levels of care for first-time fathers and stepfathers during spawn cycle 2 and 3 were similar; levels of care between experienced males, third-time fathers, and first-time fathers with prior step-fathering experience, were all similar; finally, levels of care for first-time fathers with prior step-fathering experience and first-time fathers were similar. The random effect of tank was not significant, nor was it significant for any of the analyses below.

Duration in the nest was similar for experienced males as non-breeders and first-time fathers in all groups except spawn cycle 2 where the male was removed but the female was present (Fig. 2B). This was indicated by a significant ANOVA (F_5,25_ = 3.5, p = 0.02), and post hoc tests only different for non-breeder in spawn cycle 2 versus the other groups (all p < 0.05).

**Figure 4:**
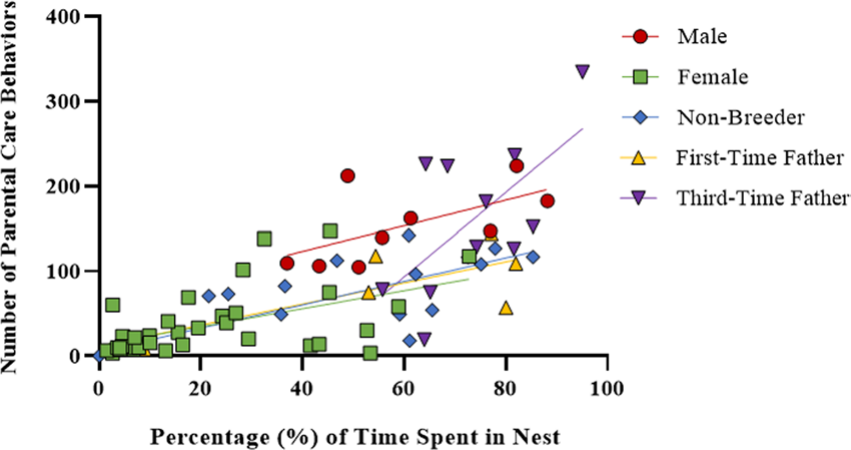
Experienced males display greater number of parental acts per unit time in the nest than all other groups. Total number of parental care acts plotted against duration in the nest for all individuals in the dataset except non-breeders in spawn cycle 1, which displayed zero time in the nest. The following groups are shown as separate symbols: males (Experiment 1), females (spawn cycles 1 and 2), stepfathers (Experiment 1- spawn cycles 2 and 3), first-time fathers (Experiment 3), and first-time fathers with previous step-fathering experience (Experiment 2) collapsed together with third-time fathers (Experiment 3) since both had the same amount of experience fathering. The solid lines represent the linear regression lines for each group.

For a given time in the nest, experienced males displayed approximately 1.8-fold more parental acts than females, non-breeders or first-time fathers. This was indicated by a positive correlation between time in the nest and number of parental care behaviors (F_1,66_ = 46.5, p < 0.0001; Fig. 4), and a significant effect of group in the analysis of covariance (F_4,66_ = 3.2, p = 0.02). The interaction between duration in the nest and group was not significant. Post-hoc pairwise differences between least-square means (after adjusting for duration in the nest) indicated experienced males were different from all groups except third time fathers (all p < 0.02), and third time fathers were different from all the rest of the groups except the stepfathers (all p < 0.04).

### Increased female parental effort when the male is removed is associated with disruption of the social hierarchy rather than partner inexperience

In the male-only removal condition (spawn cycle 2), the female displayed a higher amount of parental care as compared to all other conditions (Fig. 2A, compare all the green bars). This was indicated by a significant mixed-effects ANOVA (F_4,16_ = 11.4, p = 0.0001), and significant post hoc tests between the male removal condition as compared to all others (all p < 0.002). No other pair-wise differences were significant. The same qualitative result was found for time in nest (F_4,16_ = 12.6, p < 0.0001; Fig. 2B); only post hoc tests between the male removal and other conditions were significant (all p < 0.001). These results suggest the female entered the nest more and elevated care because the social hierarchy was disrupted rather than because the father was inexperienced.

### Egg survival increases with fathering experience and does not depend on kinship

**Figure 5:**
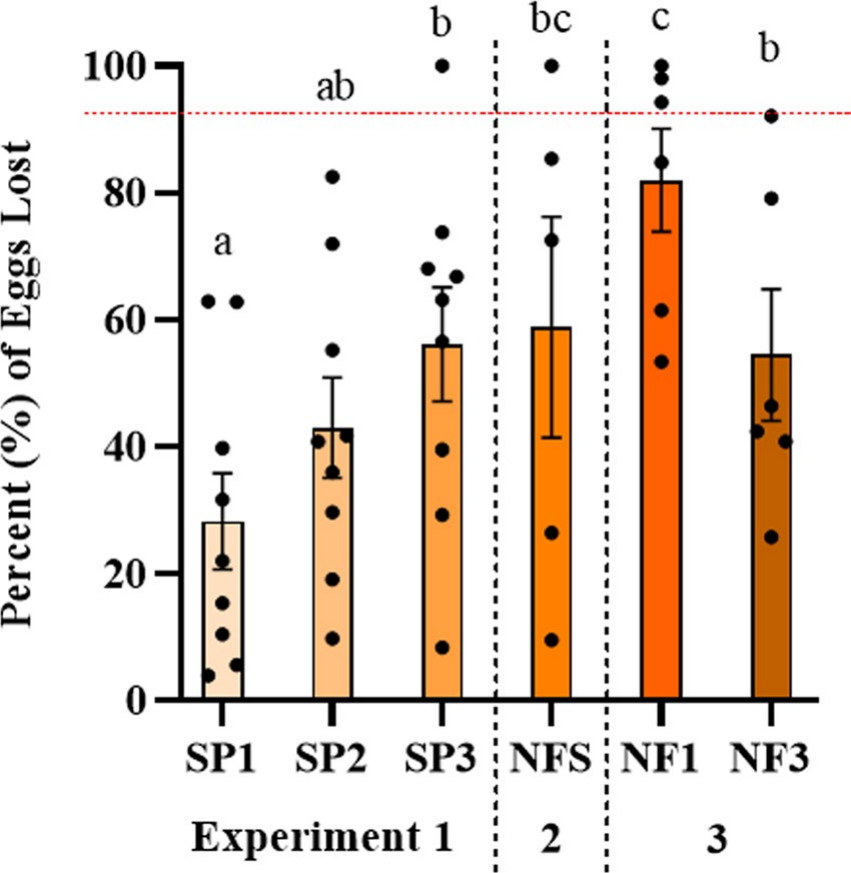
Egg survival increases with fathering experience. Mean percent egg loss (number of eggs on Day 2 minus Day 6 divided by number on Day 2) shown separately for each treatment. Different letters denote significant post-hoc differences between means. Individual data points and standard error bars are shown. The red dashed line at the top shows proportion egg loss when eggs are left alone without any individuals to tend to them. For x-axis legend see Fig. 1.

Fathering behavior was essential for survival of the eggs, as the treatment where eggs were left alone without any parenting experienced extremely high egg loss (nearly 93%; see Fig. 5 dashed red line at the top). Overall, survival rate was highest in the experienced males, and lowest in the first-time fathers, whether they were caring for eggs that were kin or not (Fig. 5). This was indicated by a significant 1-way ANOVA (F_5,25_ = 4.7, p = 0.003). Post hoc tests indicated significant differences between the first-time fathers and all other groups except first-time fathers with previous step-fathering experience (all p < 0.03). Pair-wise differences between the control condition when all 3 individuals were present (spawn cycle 1) and all other conditions were significant except when only the male was removed and female was still present (all p < 0.05).

### Experiment 4: Non-breeders with zero fathering experience

#### Non-breeders with zero fathering experience spontaneously care for eggs introduced into their territory

**Figure 6:**
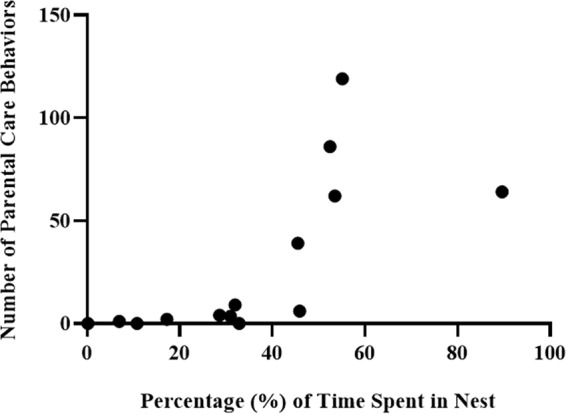
Spontaneous fathering by non-breeders after egg introduction. Total number of parental behaviors plotted against percentage time in the nest. These data are for 16 non-breeders that were housed individually for 2 weeks and then had eggs introduced into their territory for 90 min. Behaviors were scored the last 20 min of the 90 min test, and are shown as average number of behaviors per 10 min for consistency with the previous graphs.

Five out of the 16 non-breeders exposed to eggs spontaneously displayed substantial levels of parental care behaviors (i.e., nips and fans) within the last 20 min of a 90 min egg exposure (Fig. 6). Levels in these 5 individuals were similar to the stepfathers and first-time fathers in Fig. 2, noting that in Fig. 2 the behavior was measured over 4 days rather than first 90 min of exposure. A positive correlation was observed between time spent in the nest and total number of parental care behaviors (n = 16, Pearson’s *r* = 0.86, p < 0.0001; Fig. 6).

## Discussion

Here we describe for the first time what appears to be spontaneous, unconditional, and highly motivated alloparental care in *A. ocellaris*. *A. ocellaris* non-breeders cared for eggs they did not sire and which were not kin with similar effort as they cared for their own, even when the biological parents were removed from the territory thus eliminating all apparent extrinsic reasons for motivating the behavior. Most other fishes in a similar situation take advantage of the available energy by eating the eggs, rather than expend large amounts of energy caring for eggs that are not their own^31,32^. We speculate that because of their unique life history, living in small isolated groups with a strict dominance hierarchy, the probability is low that they would be in a situation where they are in a nest with eggs that are not their own. Hence, in *A. ocellaris*, the plasticity may have been lost and fathering behavior became automatic and inflexible to extrinsic factors^39^.

The evidence presented in this paper suggests that *A. ocellaris* display a hard-wired neural circuit which is activated when eggs are present that promotes fathering behavior regardless of who sired the eggs. While it is possible that the non-breeders cared for the eggs after male and female removal in Experiment 1 (spawn cycle 3) because of past experience with the female present (spawn cycle 2), Experiment 4 shows that non-breeders without any experience fathering or ever witnessing a spawning event, spontaneously care for eggs that are introduced into their territory (Fig. 6). The explanation for why only 30% and not more of the non-breeders displayed the behavior within the allotted 90 min could be related to any number of factors including individual differences in the motivational circuits, chance events such as swimming near the eggs, spending time in the terra cotta pot before eggs were presented, gonadal status or presence of steroid hormones. It is important to note here that even though fathering behavior occurred automatically without any experience in some of the individuals, it still benefits from experience, as shown by increased number of behavioral acts (Fig. 2A) and increased egg survival (Fig. 5) with successive broods. Hence, fathering in *A. ocellaris* is spontaneously initiated, but also improves with experience.

Automatic and unconditional fathering such as we observed in *A. ocellaris* is unusual in nature prompting the question of what the evolutionary origin could be. We did not directly measure fitness in this study. Therefore, we cannot definitively conclude the individuals derive zero individual or inclusive fitness benefit from the alloparental care. Nevertheless, in our opinion, the null hypothesis should be that the trait is *not* an evolutionary adaptation and the burden of proof should be establishing that it *is* an adaptation rather than the other way around^40,41^. Therefore, we favor the more parsimonious explanation that spontaneous step-fathering could have evolved in *A. ocellaris* as a side effect of selection for caring for genetic offspring. A similar spandrel hypothesis was proposed as an alternative to the adaptation hypotheses for alloparental care in birds^41^.

In some bird species, males have been observed adopting offspring of their mate mid-season if the original male is displaced. However, in this case, unlike in *A. ocellaris* when both the biological parents are eliminated, the stepfather has an obvious advantage of being paired with the female for the next breeding cycle. It therefore may either benefit from the experience (“the skills” hypothesis), or be worth the effort in order to secure a mate the following season or remain within the natal group (the so called, “pay-to-stay” hypothesis)^42–46^. The data show that *A. ocellaris* indeed do benefit from experience both in terms of number of parental behaviors (Fig. 2A) and egg survival (Fig. 5), which might on the surface support the skills hypothesis. On the other hand, the applicability of these adaptation hypotheses to the alloparental care observed in *A. ocellaris* in absence of the breeding pair is questionable. This is because if the male and female are eliminated from the territory, the third non-breeder would most likely eventually differentiate into a female, and mate with a new recruit after a period of years^14,15,24^. Hence, the experience would be for nothing, as the females are not the primary care-givers^37^ and with the male and female eliminated, the non-breeder would unlikely have an opportunity to mate in a male role. Under these conditions, to enhance individual fitness, a better strategy would seem to be for the non-breeder to consume the eggs and build somatic growth, especially since substantial growth is known to precede sex change in *A. ocellaris*^24,47^. Hence, the unconditional fathering behavior seems to, if anything, impair fitness slightly when the biological parents are displaced, which favors the spandrel hypothesis over adaptation.

Although it is relatively rare in nature for animals to care for eggs that are not kin in absence of the biological parents enforcing the rules, there are many examples where animals sneak fertilization of eggs, or place their own eggs into the nest of others to care for them, referred to as brood parasitism^48–50^. In cases of brood parasitism, the parents are caring for their own offspring first, and they cannot distinguish kin from non-kin. The behavior displayed by *A. ocellaris* does not appear to meet this definition since the non-breeders were not caring for their own eggs first. Moreover, they were present in the territory to witness the spawning event and had ample exposure to stimuli indicating the eggs were not their own such as being actively chased away from the nest.

In the previous experiment conducted by Yanagisawa and Ochi^8^, in *A. clarkii* it was concluded that the non-breeders most likely provided care for eggs due to persuasion by the female^8^. In our experiment, female levels of aggression did rise when the male was removed from the tank, consistent with Yanagisawa and Ochi’s hypothesis that the female may force the non-breeder to parent (Fig. 3A, green symbols are higher in spawn cycle 2 than 1). However, in our study, this aggression seemed to be elicited by the bold approaches of the non-breeder to the nesting area (Fig. 3B). Moreover, when both the female and male were removed, the non-breeder elevated parental care behavior (Fig. 2A, compare non-breeders in spawn cycle 3 versus 2), suggesting that the presence of the female was, if anything, a deterrent to providing care for eggs rather than a facilitator, though increased experience may also have contributed to the difference. The result that *A. ocellaris* non-breeders care for non-kin even when they are alone with the eggs in absence of any extrinsic social influences establishes that aggression by the female is not necessary for the non-breeder to display parental care, and supports the idea that parental care in *A. ocellaris* occurs unconditionally and automatically in response to the egg stimulus.

The increased aggression of the females toward the non-breeder when the male was removed, may have been related to shifts in the dominance hierarchy, rather than attempts to coerce the non-breeder to care for the eggs. Previous studies have shown that changes in social hierarchy produce increased aggression by the dominant animal in a variety of species^51–53^. *A. ocellaris* live in a strict, sized-based social hierarchy, and social ranking highly influences how individuals behave and interact with one another^13,18,52,54^. Taken together, we conclude that the heightened aggression by the female is not to compel the non-breeder to care for the eggs, but instead is a response to the change in social structure. It is possible that this female aggression is due to the female not being accustomed to the non-breeder approaching the nest, because before the male was removed, the non-breeder was treated more as an intruder and chased away from the nest by the breeding pair. It may take some time for the female to accept the non-breeder in his new ‘male’ role, and to accept that the former male is indeed gone. It is also possible these aggressive displays from the female may be signaling her place as the dominant female in the hierarchy. These are consistent with our data showing that female aggression is low and similar across conditions when the pair is alone in the tank regardless of whether the pair is established for a long time with an experienced male or a newly-formed pair with a first-time father and poorer performance, and only elevated when the social hierarchy is disrupted in the middle of a spawn cycle (Fig. 3A, compare green symbols after spawn cycle 1).

In conclusion, we found that non-breeding *A. ocellaris* are intrinsically motivated to display fathering behavior regardless of paternity or kinship. When both the male and female are removed, non-breeders care for eggs that they did not sire and which are not kin with equal effort as their own. We speculate that such highly motivated, unconditional fathering behavior may have evolved in *A. ocellaris* because of their peculiar life history in which the probability that an individual would find itself in front of eggs that are not their own is low. As the necessity for plasticity was diminished, the mechanisms to distinguish kin from non-kin were lost, resulting in automatic stereotyped fathering whenever eggs are present and the opportunity becomes available^39^. Under these conditions, we believe alloparental care in *A. ocellaris* is best described as a spandrel or side effect of selection for fathering genetic offspring rather than a de-novo adaptation in its own right^40,41^.

## Data Availability

The datasets generated during and/or analyzed during the current study will be made publically available in coordination with the journal after the paper is published.
